# Efficient Hydrolysis of Dichlorvos in Water by *Stenotrophomonas acidaminiphila* G1 and Methyl Parathion Hydrolase

**DOI:** 10.3390/ijms26199572

**Published:** 2025-09-30

**Authors:** Quyang Mei, Rimao Hua

**Affiliations:** 1 Anhui Provincial Key Laboratory of Hazardous Factors and Risk Control of Agri-Food Quality Safety, College of Resources and Environment, Anhui Agricultural University, Hefei 230036, China; 2 Institute of Ecological Environmental Protection and Pollution Remediation Engineering, Anhui Agricultural University, Hefei 230036, China; 3 Joint Research Center for Food Nutrition and Health of IHM, Anhui Agricultural University, Hefei 230036, China

**Keywords:** DDVP, *Stenotrophomonas* G1, efficient degradation, MPD, mechanism

## Abstract

Dichlorvos (DDVP) has been used in the management of agricultural pests for a long time. DDVP can cause DNA damage in mammals, and its residues in the environment and food have attracted attention. In this study, we reported a DDVP-degrading strain, *Stenotrophomonas acidaminiphila* G1, which could degrade DDVP to 20 mg/L with a DT_50_ of 3.81 min at 37 °C, a pH of 7.0, and a concentration of 1.18 × 10^10^ colony-forming units (CFUs)/mL. Strain G1’s DDVP degradation products were determined by comparison with standard substances and UPLC-MS/MS analysis. The results showed that dimethyl phosphate (DMPP) was the main metabolite of DDVP, and its toxicity to non-target organisms was significantly lower than that of the parent compound. Furthermore, the key genes for the degradation of DDVP by strain G1 were analyzed using whole-genome sequencing. A methyl parathion hydrolase gene, *mpd*, was identified, and its activity was verified through prokaryotic expression and enzyme kinetics. The purified enzyme MPD could entirely degrade 20 mg/L DDVP within 1 min. These results not only provide biological resources for the rapid degradation of organophosphorus pesticides but also offer a theoretical basis for the efficient remediation of pesticide residues.

## 1. Introduction

Organophosphorus pesticides (OPs) are one of the most widely used insecticides worldwide [1]. Due to their high toxicity and the detection rate in environmental and agricultural product samples, they have attracted extensive attention [2]. Although some highly toxic OP varieties have been banned in developed countries, they are still widely used in developing countries. For example, dichlorvos (DDVP) was classified as a class 1B “highly hazardous” pesticide by the World Health Organization (WHO) in 1992, and the U.S. Environmental Protection Agency (EPA) also classified it as a highly toxic compound [3,4]. However, it is still one of the main OP varieties for controlling household sanitary pests and agricultural pests in developing countries today [5,6]. Furthermore, DDVP residues pose a high risk to the environment and agricultural products. A study detected pesticide residues in 29 samples from seven major coix seed-producing areas in China, among which DDVP had the highest residual risk [7]. Studies determined the pesticide residues in water samples from the Llobregat and Ter basins in Catalonia, Spain. Among them, the residue of DDVP exceeded the limit standard with high risk (hazard quotient > 10) [8]. DDVP residues not only harm the environment but also pose a potential threat to human health.

DDVP mainly causes the death of target insects by inhibiting the activity of acetylcholinesterase [9]. At the same time, this effect is also harmful to mammals. It can be ingested, inhaled, and absorbed through the skin, and can penetrate biological barriers and accumulate in the cytoplasm [10]. Studies have found that in addition to neurotoxicity, DDVP also has hepatotoxicity, nephrotoxicity, and genotoxicity [11,12]. Epidemiological studies showed that exposure to DDVP can increase the incidence of liver diseases. In vivo and in vitro model experiments on rats also found that DDVP can affect the ABC transporters and amino acid biosynthesis in rat liver cells [13]. Furthermore, DDVP can reduce sperm cells in male rats through oxidative stress and cause testicular tissue lesions [14]. Therefore, finding a rapid degradation method for DDVP residues is of great significance for environmental and ecological risks and human health.

Photolysis and biodegradation are the main ways to remove DDVP residues in the environment [15]. However, in actual remediation, to improve the efficiency of photodegradation, additional catalysts are often required, which may cause secondary pollution [16]. In contrast, degrading functional microorganisms screened from environmental samples have become a better choice [17]. In recent years, some microorganisms with the ability to degrade DDVP have been screened and used for the actual remediation of environmental pollution and detoxification after DDVP exposure poisoning. *Pseudomonas stutzeri* smk can grow with DDVP as the sole carbon source, and it can degrade 80% of DDVP at 30 °C within 7 d [18]. The degradation activity of *Trichoderma atroviride* T23 is affected by the initial concentration of DDVP and the type of culture medium. The intracellular enzymes produced in strain T23 on a PDA medium can degrade more than 90% of 100 μg/mL DDVP within 72 h [19]. Feeding with *Lactobacillus plantarum* CCFM8661 can significantly alleviate the oxidative stress and inflammatory response caused by DDVP poisoning in mice [20]. However, the degradation activities of these degrading strains are limited, especially in the actual complex environment. Meanwhile, more studies have focused on the microbial degradation activity of DDVP and the determination of its metabolites, while relatively few studies have been conducted on the degradation functional genes of DDVP and the catalytic molecular mechanism of degradation enzymes [21]. This further restricts the optimization of the structure of the degrading enzyme to obtain a DDVP-degrading enzyme with higher catalytic activity and stronger environmental adaptability.

Therefore, we isolated a DDVP-degrading strain, *Stenotrophomonas acidaminiphila* G1. The degradation half-life of DDVP by strain G1 is only 3.81 min, which is the fastest reported DDVP-degrading strain so far. On this basis, we systematically studied: (1) the degradation kinetics and characteristics of DDVP by strain G1; (2) analyzed the biodegradation products of DDVP; (3) identified the key genes involved in DDVP degradation by strain G1 using genomics; and (4) expressed and purified the degrading enzyme and analyzed the catalytic mechanism of the degrading enzyme for DDVP hydrolysis. The results provide a new solution strategy for the rapid removal of DDVP residues and pollution remediation in environmental and agricultural product samples.

## 2. Results

### 2.1. Metabolism of DDVP by Strain G1

**Figure 1:**
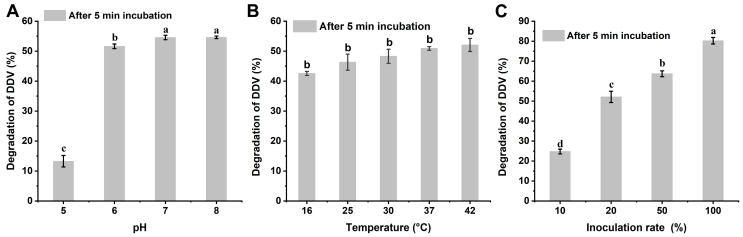
Degradation rate of 20 mg/L DDVP by strain G1 under different pH levels (**A**), temperatures (**B**), and inoculation amounts (**C**) within 5 min. (The data are represented as the mean ± standard deviation for triplicate. A–d letters are marked based on difference significance analysis).

A total of 20 mg/L of DDVP could be degraded by strain G1 at a pH of 7 and a temperature of 37 °C. The degradation curve was well fitted with the first-order kinetic equation (R^2^ > 0.95). The DT_50_ of DDVP was only 3.81 min. We further determined the influencing factors of DDVP degradation by strain G1. The degradation rates of DDVP were 13.3%, 51.6%, 54.6%, and 54.6% at pH levels of 5, 6, 7, and 8, respectively, after 5 min (Figure 1A). The optimum pH for DDVP degradation was between 7 and 8. Figure 1B shows the degradation of DDVP by strain G1 at different temperatures (16, 25, 30, 37, and 42 °C), with degradation rates of 42.6%, 46.3%, 48.3%, 50.9%, and 52.1% at 5 min, respectively. The optimum temperature for DDVP degradation by strain G1 was between 25 and 42 °C. Figure 1C showed a proportional relationship between the degradation rates and the G1 inoculation amounts at pH 7 for 5 min.

**Figure 2:**
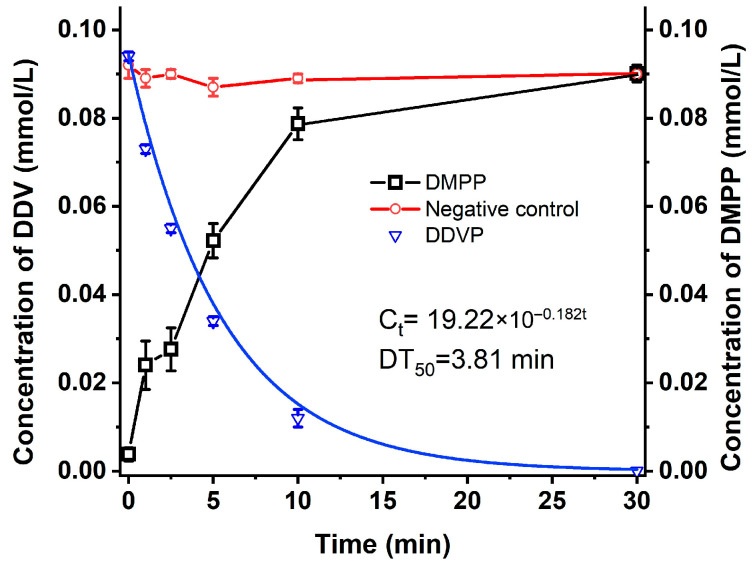
Degradation kinetics of DDVP and DMPP by strain G1. (pH 7, 37 °C, incubation amount 20% (OD_600_ = 1.0), initial concentration 20 mg/L); red line: 20 mg/L DDVP in MSM medium without strain G1; black line: DMPP generated from 20 mg/L DDVP in MSM medium with strain G1; blue line: DDVP residual in MSM medium with strain G1.

The metabolite of DDVP, produced by strain G1, was also detected by UPLC, and a metabolite, DMPP, was measured and identified (Figure S1). Strain G1 could metabolize DDVP to DMPP as the sole metabolite but was unable to degrade its metabolite, DMPP (Figure 2).

### 2.2. Genome Features of Strain G1 and Degradation Gene in Strain G1

To analyze the molecular mechanism of DDVP degradation by strain G1, we determined the whole-genome sequence of strain G1 through genome sequencing. A total of 153,699 reads were obtained and sequenced. The mean read length and N50 read length were 11,725 bp and 14,657 bp, respectively. The sequencing depth and coverage were 450× and 100%. After assembly, the full length of the genome was 4,016,985 bp, and it consisted of only one circular chromosome with a G+C content of 69% (Table S1). A total of 3674 coding sequences (CDSs) were identified within the genome, and 2331 genes were assigned functional annotations, of which 1236 genes exhibited enzymatic catalytic activity in the GO database (Figure S2).

In strain G1, DDVP was primarily metabolized through the hydrolysis of the P-O bond, which should be catalyzed by phosphate ester hydrolase. We screened genes related to phosphatase activity in the annotation of strain G1 genome, and 21 phosphatase genes were identified in the KEGG database, including phosphotriesterase and phosphodiesterase (Table 1). Among these genes, a methyl-parathion hydrolase gene (*mpd*, G1_GM001006) has attracted attention, as it has been reported to have the ability to degrade a variety of organophosphorus insecticides.

**Figure 3:**
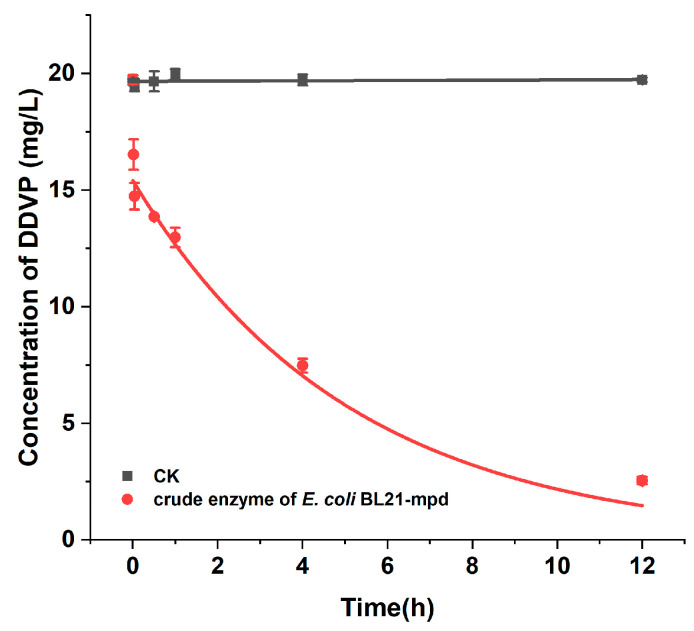
Degradation of 20 mg/L DDVP by crude enzyme from an engineered strain.

To verify the functional characteristics and catalytic properties of the *mpd* gene, we generated a recombinant strain to express the enzyme and assessed its capability to degrade DDVP. The *mpd* gene was cloned into the pMD19-T vector and transferred to the *E. coli* DH5α competent cells. The positive clone was isolated, and its degradation ability was measured. The results were shown in Figure 3. DDVP could be degraded by mpd-positive clone cells, and the DT_50_ of DDVP was only 7.2 min. The results indicated that the *mpd* gene was the key gene for DDVP degradation in strain G1.

### 2.3. Degradation Kinetics of Purified Enzyme MPD for DDVP

**Figure 4:**
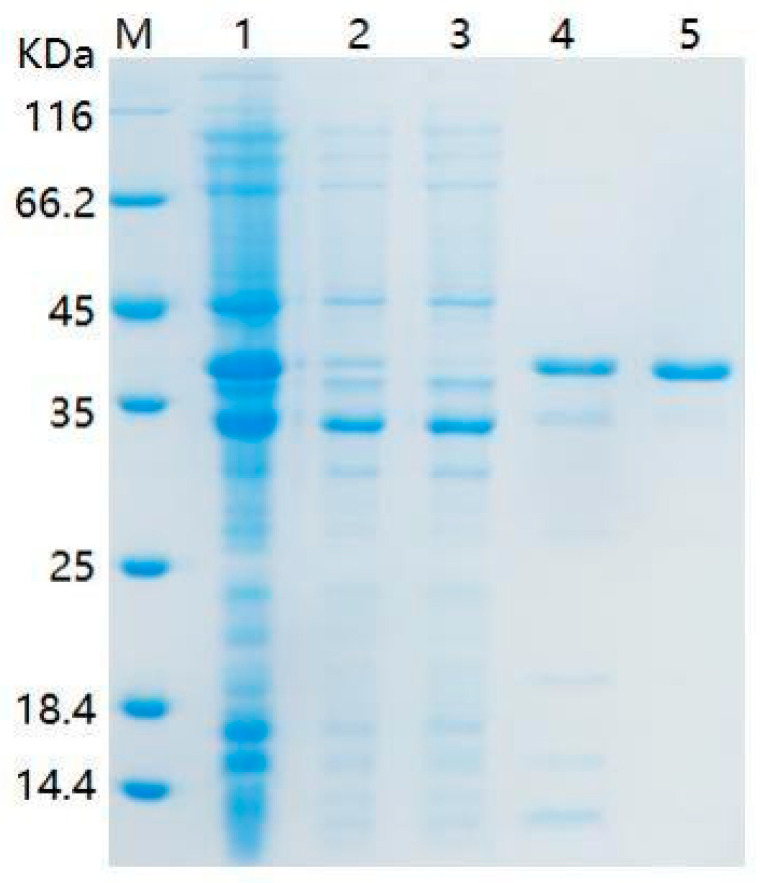
The SDS-Page of crude enzyme and purified MPD. Line M: Marker; Line 1: IPTG induced; Line 2: supernatant; Line 3: effluent; Line 4: 50 mM imidazole eluent; Line 5: 500 mM imidazole eluent.

**Figure 5:**
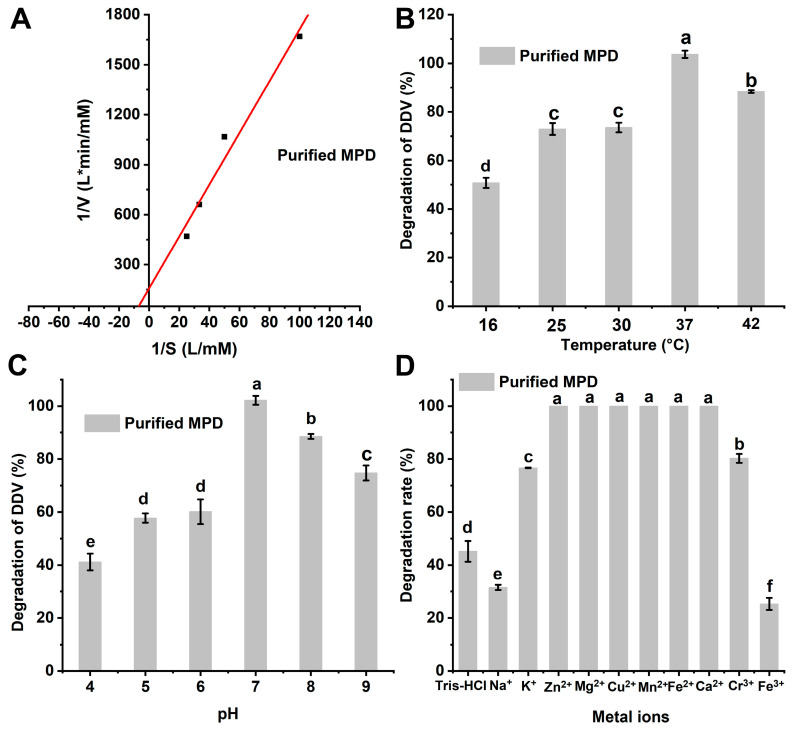
Michaelis constant of enzyme MPD (**A**); degradation characteristics of enzyme MPD under different temperatures (**B**), pH levels (**C**), and metal ions (**D**). (The data are represented as the mean ± standard deviation for triplicate. a–f letters are marked based on difference significance analysis).

After determining the activity of the *mpd* gene, we constructed an expression vector and purified the expressed degradation enzyme MPD using a Ni-affinity chromatography column (Figure 4). The degradation kinetic constants of enzyme MPD were also measured. The results showed that 150 mg/L purified MPD could entirely degrade 20 mg/L DDVP within 1 min. The data were well fitted to the Michaelis–Menten equation (R^2^ = 0.9563). The Vmax and Km of purified MPD were 6.37 × 10^−3^ mmol/L·min and 0.0990 mmol/L, respectively (Figure 5A).

The degradation characteristics of purified MPD under different temperatures, pH levels, and metal ion conditions were investigated in Figure 5B–D. The degradation rates of DDVP by MPD at 16, 25, 30, 37, and 42 °C were 50.8%, 73.0%, 73.6%, 100%, and 88.3%, respectively, indicating that the optimal temperature for MPD was 37 °C. At different pH values, the catalysis efficiency of purified MPD varied significantly (*p* < 0.05). MPD exhibited the highest degradation activity at a pH level of 7.0, with a degradation rate of 92.9% for 20 mg/L DDVP in 1 min.

The degradation enzyme MPD also displayed great differences under varying valence metal ions. As shown in Figure 5D, divalent metal cations (Zn^2+^, Mg^2+^, Cu^2+^, Mn^2+^, Fe^2+^, and Ca^2+^) promoted the degradation of purified MPD. A noticeable difference was seen (*p* < 0.05) under the conditions of K^+^ and Na^+^ during the DDVP degradation process. K^+^ promoted the activity of MPD, while Na^+^ inhibited it. Cr^3+^ promoted the activity of MPD, whereas Fe^3+^ inhibited it.

### 2.4. Catalytic Mechanism of Degrading Enzyme MPD on DDVP

**Figure 6:**
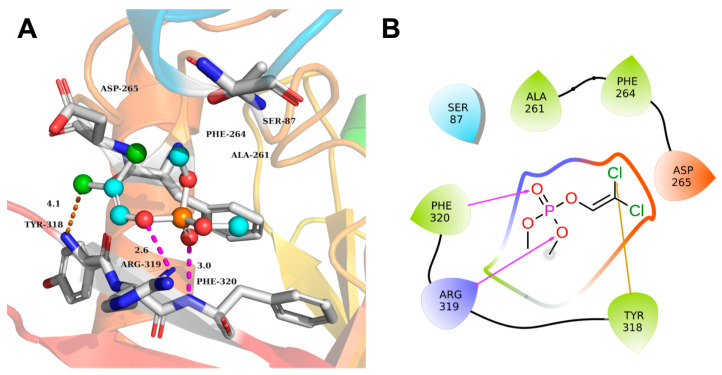
Molecular docking of DDVP with enzyme MPD (**A**); the binding mode diagram of DDVP and MPD (**B**). (The yellow and purple lines represent hydrogen bonds and halogen bonds respectively, and the numbers on the lines indicate the bond lengths).

To analyze the degradation and catalytic mechanism of the degrading enzyme MPD on DDVP, we used the AlphaFold2 model to construct the 3D structure of enzyme MPD and determined the binding sites through molecular docking. The results showed that the binding energy between DDVP and MPD was −5.24 kcal/mol (Table 2). DDVP could interact with the active pocket (comprising Ser87, Ala261, Phe264, Asp265, Tyr318, Arg319, and Phe320) in MPD through hydrogen bond and halogen bond (Figure 6). Phe320 and Arg319 formed hydrogen bonds with the O (on the phosphate group) of DDVP, respectively. Try318 formed halogen bonds with the Cl (on the phosphate group) of DDVP.

**Figure 7:**
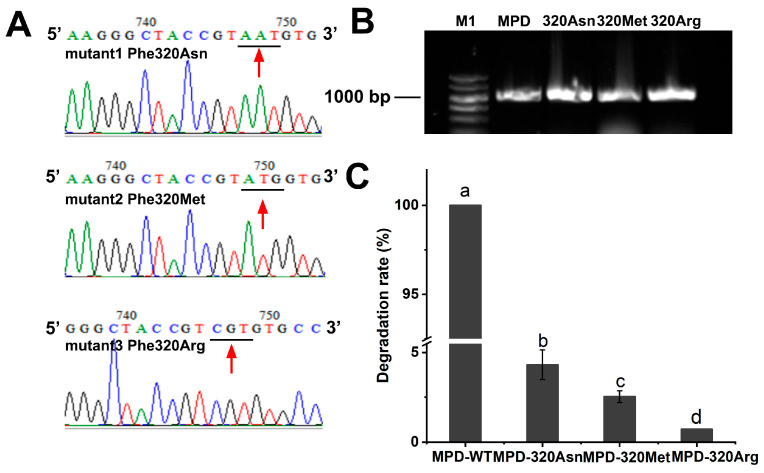
(**A**) Sequencing chromatogram of the mutants; (**B**) gel electrophoresis of the mutant Phe320Asn, Phe320Met, and Phe320Arg; and (**C**) degradation of DDVP via wild-type MPD and mutants. (The data are represented as the mean ± standard deviation for triplicate. a–d letters are marked based on difference significance analysis).

The molecular docking analysis revealed that the ester bond served as a hydrogen bonding site for Phe320, indicating its potential function as a key interaction site. The finding suggested that Phe320 may play a crucial structural or catalytic role in the molecular recognition process; therefore, we mutated Phe to Asn, Met, and Arg and compared the degradation ability of DDVP by the wild-type MPD and the mutants Phe320Asn, Phe320Met, and Phe320Arg (Figure 7). The wild-type MPD could completely degrade 20 mg/L DDVP within 5 min, whereas the mutants Phe320Asn, Phe320Met, and Phe320Arg were unable to degrade DDVP. These results indicated that Phe320 was the key amino acid site for MPD to catalyze the breakage of the phosphate ester linkage of DDVP.

## 3. Discussion

DDVP is an organophosphorus phosphate insecticide with high water solubility, which has been widely used in agriculture and poses a threat to fish, mammals, and humans [22]. The toxicity is mainly caused by inhibition of acetylcholine esterase, an enzyme crucial for the nervous system of organisms [18]. The DT_50_ of DDVP ranges from 10.5 to 19.8 d in soil [23]. Microbial degradation is recognized as an effective, low-cost, and high-efficiency method for eliminating DDVP pollution [18,19,24]. We found that strain G1 was identified as an efficient strain in the degradation of DDVP; pH level, temperature, and incubation time were found to affect the degradation by strain G1.

Strain G1 showed high efficiency when it was compared with other DDVP degradation strains (Table 3). *Pseudomonas* AUG12 could grow in 1500 mg/L of DDVP and degrade 100 mg/L of DDVP in 140 h; the degradation rate was 1.435 mg/L·h [25]. *Ochrobactrum* sp. DDV-1 could completely degrade 100 mg/L DDVP within 24 h [26]. Strain G1 was the most efficient bacterium for DDVP degradation, with a DT_50_ of 3.81 min. The DT_50_ of *Flavobacterium* YD-4 and halophilic bacteria T10/1 were 245 and 69 times greater than that of strain G1 [24,27]. Even under high-concentration conditions, strain G1 can still maintain high degradation activity. We further determined the degradation kinetics of strain G1 for 200 mg/L and 500 mg/L DDVP. The DT_50_ of 200 mg/L and 500 mg/L DDVP by strain G1 were only 18.24 min and 46.20 min, respectively (Table S3).

In addition, some bacterial strains could degrade DDVP to other toxic metabolites. *Trichoderma atroviride* T23 could degrade DDVP to 2,2-dichloroethanol and 2,2-dichloroacetate [31]. *Stutzeri* smk could produce degradation intermediates, including dimethyl vinyl phosphate, 2-chloroacrylyl dimethyl phosphate, dimethyl phosphate, and methyl phosphate, during the biodegradation process [18]. d-DDVP was produced, which proved to be more toxic than DMPP [32]. Dichloropropanol residues were found among the degradation products of DDVP [33]. Except for DMPP, dimethyl phosphate and trimethyl phosphate were also identified among the products [34].

Unlike previous studies that generated persistent toxins, strain G1 could degrade DDVP to the sole metabolite DMPP (LC_50_ for mice is 8714 mg/kg) that was not toxic to non-target organisms [35]. The strain exhibited a “clean” metabolic profile, minimizing secondary pollution risks. We observed that no other products were produced except for DMPP during DDVP degradation by strain G1. This is of great benefit in practical applications, suggesting the environmental safety of this bacterial strain and the potential for eco-friendly applications. Effective detoxification could be achieved through the biodegradation of strain G1, thereby reducing environmental residual risks. While current results demonstrated an absence of harmful byproducts, large-scale field trials are needed to rule out context-dependent metabolite generation. Meanwhile, we also measured the degradation activity of strain G1 against various pesticides. The results showed that, in addition to DDVP, strain G1 could also efficiently degrade methyl parathion, parathion, chlorpyrifos, fenitrothion, phoxim, triazophos, thiram, and profenofos (Table S4). This characteristic enables strain G1 to be applied in the remediation of environments contaminated with various pesticide residues.

Enzymes primarily catalyze the biodegradation of contaminants. Some genes were responsible for the degradation of DDVP, including *mpd*, *opd*, *hph* [35], cytochrome P450 [31], Tapon1-like [36], and *aph* [37]. However, the gene responsible for DDVP degradation in strain G1 was not clear. We searched for the related degradation genes of DDVP by sequencing and analyzing the genome of strain G1. Genomic analysis revealed that the strain G1 utilizes an enzyme, MPD, which can degrade DDVP into DMPP, thereby preventing the accumulation of other hazardous metabolites. The strain’s MPD enzymes ensured the breakdown of DDVP into non-toxic DMPP molecules, thereby preventing the formation of harmful intermediates. In addition, by comparing the reported DDVP-degrading genes in the NCBI database, we found that the key degrading gene of strain G1 had the highest homology with the *mpd* gene from the strain WBC-3. Instead of being on a plasmid, the *mpd* gene was located on the chromosome, indicating that the degradation gene was conserved in strain G1. Additionally, there were two regulatory factors, *arsR* and *arsC*, downstream of the *mpd* gene. According to the literature reported, transcription regulators can regulate the expression of degradative genes for exogenous substances such as aromatic compounds. After the induction of exogenous substances, transcription regulators can enhance the expression of genes encoding degrading enzymes [38]. In addition, strain G1 exhibited robust xenobiotic metabolic capabilities. Whole-genome analysis revealed 17 CDSs associated with aromatic compound metabolism, along with functional genes for heavy metal degradation, highlighting its potential for bioremediation in complex polluted environments.

According to the genome sequence, 21 phosphate genes were annotated in strain G1; the *mpd* gene was identified as the key degradation gene of DDVP. There were conserved sequences with higher homology in MPD compared with MPH (1P9E_A) from *Pseudomonas* WBC and MBL (WP_080708177). MPD contained a serine residue, a typical catalytic active site for metalloenzymes, and a Ser-His-Asp/Glu triad, which is a hallmark of α/β hydrolases. Conserved amino acids were found in the sequences of these enzymes. They all contained four residues in the H-X-H-X-DH sequences, which were involved in metal coordination (residues 157 to 162) (Figure S3).

MPD had the highest similarity with the metal folding enzyme MBL from Pseudomonas and methyl parathion hydrolase (MPD, EC 3.1.8.1) from Pseudomonas WBC-3, with a similarity of 100%. MPD, a Zn^2+^-containing enzyme, catalyzes the degradation of the organophosphorus pesticide methyl parathion. The results of crystal structure analysis showed that MPD has a dimeric structure with 331 amino acids per monomer [39]. Class B beta-lactamases have broad-spectrum substrate characteristics, which are associated with bacterial resistance to penicillin. The amino acid sequence comparison of MPD with BBLs showed that the amino acid sequence similarity between BBLs and MPD was less than 30%, but five of the six potential zinc-binding residues were conserved [40]. Zn^2+^, Mg^2+^, Cu^2+^, Mn^2+^, Fe^2+^, Ca^2+^ and Cr^3+^ could enhance the activity of MPD, while Na+ and Fe3+ inhibited its activity. The crystal structure showed that the presence of two metal cations in the active center significantly affected the degradation efficiency. The activities of the enzyme and substrate rely on the interaction between the substrate and divalent cations [41]. The optimum temperature, pH level, and conditions of OPHC2 with methyl parathion were 65 °C. As MPD was a metalloenzyme with a bivalent metal ion at its active site, the influence of exogenous metals in the environment on their enzymatic activity and the practical efficacy of the bacterial strain required further investigation.

Studies were conducted to clarify the catalytic mechanism of MPD when it interacted with the substrate. In the connection between MPD and methyl parathion, the histidine and tyrosine in MPD promote substrate binding through π-π stacking interactions [42]. The active pocket (Ser87, Ala261, Phe264, Asp265, Tyr318, Arg319, and Phe320) for DDVP degradation was adjacent to the MPD binding site. DDVP interacted with MPD through hydrogen bonding and halogen bonding interactions. The free binding energies between MPD and DDVP were exergonic, indicating that the interaction was spontaneous [43]. Molecular docking simulations revealed that MPD formed a stable complex with DDVP through hydrogen bonding and halogen bonding, with a calculated binding energy of −5.24 kcal/mol, indicating a high binding affinity. DDVP’s phosphate group formed hydrogen bonds with Phe320 (distance: 3.0 Å) and Arg319 (distance: 2.6 Å), while its Cl interacted with Try318 via halogen bonding. The docked conformation positioned DDVP’s reactive group within the catalytic amino acid, facilitating subsequent hydrolysis.

Previous studies have shown that Phe was involved in the binding process of DDVP in the degradation of methyl parathion. We found that during the binding process of DDVP and MPD, Phe, as a conserved hydrophobic residue, formed a hydrophobic cavity with DDVP. Therefore, we designed a targeted mutation at Phe to verify its key role in the degradation process of DDVP. Site-directed mutagenesis of Phe320 to Asn, Met, and Arg reduced the degradation efficiency of DDVP, validating its role in docking predicted by our model. Engineering the pocket (e.g., Phe320Arg mutation) could change the size, shape, or charge distribution of the binding pocket, which affects the entry or orientation of the substrate, thus changing the catalytic ability of enzymes. Future work should integrate QM/MM calculations to precisely model the electron transfer processes during the catalytic step. Targeted amino acid substitutions allow for the tailored optimization of enzyme activity, facilitating the design of high-performance microbial biocatalysts for environmental remediation.

## 4. Materials and Methods

### 4.1. Chemicals and Materials

DDVP (purity > 95%) was purchased from Dr. Ehrenstorfer (Augsburg, Germany); dimethyl phosphate (purity > 95%) was purchased from AccuStandard (New Haven, CT, USA). Restriction endonucleases (EcoR I and Hind III), T4 DNA ligase, and Taq DNA polymerase were purchased from Takara Biotech Company (Dalian, China).

### 4.2. Strains, Plasmids, and Growth Conditions

Strain G1 was isolated from activated sludge of an insecticide wastewater treatment facility in Jiangsu, China. Optimal growth conditions were 37 °C and a pH of 7 in a Luria–Bertani (LB) medium. *E. coli* BL21(DE3) and *E. coli* DH5α competent cells, pMD19-T, and the pET28a vector for degradation gene cloning and expression were purchased from Takara Biotech Co. (Dalian, China).

### 4.3. Degradation of DDVP by Strain G1

Strain G1 was activated and incubated in LB medium to an optical density at 600 nm (OD_600_) of 1.0 (~5.9 × 10^10^ CFU/mL). To evaluate the degradation capability of strain G1, MSM was supplemented with 20 mg/L DDVP as the sole source of carbon and energy. A 1.0 aliquot of strain G1 suspension was added to 4 mL of MSM in a 20 mL test tube. All samples in triplicate were incubated at 37 °C and 150 rpm. MSM inoculated without strain G1 was set as the negative control. To terminate the reaction and extract residual DDVP, a 5 mL aliquot of pure acetonitrile was added to each tube. All samples were sampled and analyzed to measure DDVP and its metabolites.

To evaluate the degradation of DDVP by strain G1, the effects of inoculum amount, pH, and temperature were examined. We determined inoculation amount levels of 10%, 20%, 50%, and 100% under ambient conditions of 37 °C and pH 7. The pH was adjusted to values of 5, 6, 7, 8, and 9 under the 20% inoculation amount and 37 °C. The effect of temperature was assessed at 16, 25, 30, 37, and 42 °C, with a pH of 7 and a 20% inoculation rate.

### 4.4. Complete Genome Sequencing

We used the TIANamp Bacteria DNA Kit (Tiangen Biotech Company, Beijing, China) to extract the genomic DNA of strain G1. The total DNA content was assessed through agarose gel electrophoresis and a nucleic acid protein detector. Large-scale parallel massive parallel sequencing (MPS) was conducted on samples that met the necessary sequencing criteria, employing the Illumina sequencing platform. During the sequencing process, two types of libraries were constructed: a conventional 500 bp small fragment library and a 5 kb large fragment library. Qualified DNA samples were randomly fragmented using ultrasonic treatment to generate DNA fragments of the requisite size, and an A tail was appended to the 3′ end of the fragments to complement the T base at that terminus. Subsequently, libraries of sufficient quality were constructed for cluster preparation and sequencing. Low-quality read fragments in the clean data generated from sequencing were filtered and assembled using SOAPdenovo (version 2.04) to produce a refined genomic map; however, gaps remained that required further filling. Primers were designed to perform polymerase chain reaction (PCR) using known highly homologous bacterial genome sequences and contigs derived from de novo sequencing. The specific bands obtained were sequenced, allowing for the resolution of gaps in the refined genomic maps, ultimately yielding a complete genomic representation of strain G1.

### 4.5. Prediction and Annotation of DDVP Degradation Genes

We used six databases to analyze the complete genome sequence of strain G1. Functional annotation was conducted using GO, Swiss-Prot, NR, KEGG, COG, and TCDB. Bioinformatics-based comparative annotation analysis was carried out to identify genes involved in DDVP degradation.

### 4.6. Cloning, Expression of the mpd Gene, and Purification of Enzyme MPD

The *mpd* gene was cloned from strain G1 genomic DNA by the PCR method with the primer pairs *mpd*-F and *mpd*-R. The primers were as follows: cgcggatccGAATTCATGCCCCTGAAGAACCGCTTGCTG (*mpd*-F) and gcggccgcAAGCTTgTCACTTGGGGTTGACGACCGAGTAG (*mpd*-R). EcoR I and Hind III restriction sites were added to primer pairs. The PCR procedure was as follows: polymerase was activated at 98 °C for 3 min, followed by 98 °C for 15 s, 58 °C for 15 s, and 34 cycles at 72 °C for 60 s. Then, the PCR products were ligated to the T-vector to construct pMD-*mpd* and transferred to *E. coli* BL21 competent cells. After sequencing, the plasmids pMD-*mpd* were then gel-purified and double-digested with EcoR I and Hind III restriction endonuclease. The fragments were ligated into the pET-28a (+) vector, and the expression vector pET28a-*mpd* was obtained. The constructed expression vector pET28a-*mpd* was introduced into *E. coli* BL21(DE3) competent cells, and positive clones, designated *E. coli* BL21-*mpd*, were screened on plates containing 100 mg/L kanamycin. After sequencing verification, the positive clone was cultured in 100 mL of LB medium at 37 °C until the optical density at 600 nm (OD_600_) reached 0.8. Subsequently, 1 mM IPTG was added to the medium, and the cells were further incubated at 37 °C in a rotary shaker set at 220 rpm for the overexpression of MPD. After 4 h of incubation, the cells were harvested and resuspended in Tris-NaCl buffer (20 mM Tris-HCl, 500 mM NaCl, pH 8.0). The cells were then disrupted using an ultrasonic homogenizer (Scientz-IID, Ningbo Scientz Biotech Co., Ltd., Ningbo, China) and centrifuged at 10,000× *g* for 10 min at 4 °C. The precipitated fraction was washed, collected, and re-dissolved in denaturing buffer (20 mM Tris-HCl, 8 M urea, pH 8.0). MPD in solution was then bound to a Ni-NTA resin column and washed with wash buffer (20 mM Tris-HCl, 8 M urea, 20 mM imidazole, pH 8.0). Finally, the purified MPD was eluted using elution buffer (20 mM Tris, 8 M urea, 500 mM imidazole, pH 8.0) and subsequently renatured in Tris-NaCl buffer via dialysis.

### 4.7. Determination of Enzyme Activity

We used the Linewear–Burk method to measure the Michaelis constant (Km) of MPD with the DDVP concentrations from 0.01 to 0.04 mM, and we determined the enzyme kinetic parameters by nonlinear curve fitting to the Michaelis–Menten equation. The reaction solution contained 50 µL of purified MPD (150 mg/L) and concentrations of DDVP of 0.01, 0.02, 0.03, and 0.04 mM, respectively. After 1 min incubation at 37 °C, the solution was sampled. Then, the DDVP concentrations were measured, and the results were fitted with the Michaelis–Menten equation to calculate the Km. Each treatment had three replicates.

### 4.8. Degradation Characteristics of DDVP by Purified Enzyme

Under varying temperatures (16–42 °C), pH levels (4–9), and metal ions (Ag^+^, K^+^, Na^+^, Ca^2+^, Mg^2+^, Mn^2+^, Zn^2+^, Cu^2+^, Fe^2+^, Cr^3+^, and Fe^3+^), the degradation of DDVP by purified MPD was assessed. A total of 50 μL of pure MPD enzyme, with a final concentration of 150 mg/L, was sequentially added to the standard solution of DDVP to achieve a concentration of 10 mg/L. Subsequently, Tris-HCl (pH = 7.0) was added to achieve a final volume of 5 mL. Samples lacking MPD served as negative controls. After incubation at 37 °C, samples were collected at 0 min and 1 min, and then 5 mL of acetonitrile was added to halt the reaction and extract the DDVP. The residual DDVP was subsequently analyzed using ultraperformance liquid chromatography (UPLC).

### 4.9. Analytical Methods

Metabolites of DDVP were separated by an Acquity UPLC BEH C_18_ column (1.7 μm, 2.1 mm × 100 mm) with the injection volume 10 μL and the flow rate 0.25 mL/min. The mobile phase consisted of solvent A (5‰ phosphoric acid in water) and solvent B (0.1% methanol). The separation was achieved using the liquid chromatography gradient program at 40 °C: 0–2.00 min, 5% B; 2–15.00 min, from 5% to 20% B; 15–18 min, from 20% to 95% B; 18.00–18.10 min, from 95% to 5% B; 18.10–20 min, 5% B. The capillary and cone voltages were 1500 V and 20 V, respectively. The mass spectrometer was operated in electrospray ionization (ESI) mode, with scans ranging from *m*/*z* 30 to 225. The mobile phase employed a gradient elution method, collecting both positive and negative ions.

DDVP and DMPP were quantitatively analyzed as shown in Table S2. The parent ion of DMPP was detected at *m*/*z* 221.00, with one of its daughter ions at *m*/*z* 109.00, having a dwell time of 0.05 s and cone and capillary voltages of 28 V and 22 V, respectively. Additionally, another daughter ion was detected at *m*/*z* 79.00, with the same dwell time and cone/capillary voltages of 28/34 V, respectively. The parent ion of DDVP was recorded at *m*/*z* 125.03, with daughter ions at *m*/*z* 109.00 and 62.95, with cone and capillary voltages set to 22 V and 14 V, respectively, and a dwell time of 0.05 s.

### 4.10. Homology Modeling and Molecular Docking of DDVP with MPD

The structure of the enzyme MPD was constructed via the AlphaFold2 model. Initially, hydrogenation was performed using UCSF Chimera (version 1.19), followed by the calculation of the atomic charge of the protein using the AMBER14SB force field. Configuration optimization was achieved utilizing the MMFF94 force field, resulting in the selection of the low-energy conformation. Local charge assignments were made using the AM1-BCC method within UCSF Chimera.

### 4.11. Point Mutation

To ensure the active sites, point mutations were performed by modifying the amino acid residues in the binding pocket according to the results of molecular docking. Site-specific mutations were generated using the overlap PCR method. Phe320 of MPD was replaced by Asn, Met, and Arg through site-directed mutagenesis by PCR with different primers (Table S2). Phe320Asn, Phe320Met, and Phe320Arg were generated with the primers Asn-F/Asn-R, Met-F/Met-R, and Arg-F/Arg-R by conventional PCR. We used the pET-28a-MPD plasmid as the template and performed PCR under the following conditions: 95 °C for 3 min, followed by 30 cycles of 95 °C for 15 s, 58 °C for 15 s, and 72 °C for 18 s, with a final extension step of 6.5 min at 72 °C. Subsequently, the enzyme activity of the mutant variants was determined using the same method in Section 4.6.

## 5. Conclusions

In conclusion, strain G1 could efficiently degrade DDVP in water. The sole detoxification metabolite DMPP was formed during the degradation process. The degradation enzyme MPD, encoded by the *mpd* gene, was responsible for catalyzing the degradation of DDVP. Purified MPD could degrade DDVP. DDVP was stably bound within the MPD cavity primarily through hydrogen bonding and halogen bonding interactions. Therefore, the strain G1 is an efficient candidate for the remediation of DDVP pollution in water.

## Figures and Tables

**Table 1:** Phosphatase genes in the genome of strain G1. (“+” and “−” indicates the sense and antisense strand, respectively.)

Gene Number	Locus	Annotation
G1_GM000376	Chr1:451815:452579:+	c-di-GMP-specific phosphodiesterase class I
G1_GM000403	Chr1:479899:482832:+	Sensor domain-containing phosphodiesterase
G1_GM000617	Chr1:725113:726333:−	2′3′-cyclic phosphodiesterase
G1_GM000878	Chr1:1012855:1014471:−	Phosphodiesterase
G1_GM001006	Chr1:1174990:1175985:−	Methyl-parathion hydrolase *mpd*
G1_GM001037	Chr1:1214103:1214729:−	c-di-GMP phosphodiesterase class II
G1_GM001072	Chr1:1257629:1259686:−	CdpA cyclic di-GMP phosphodiesterase
G1_GM001177	Chr1:1376461:1377048:+	RNA2′,3′-cyclic phosphodiesterase
G1_GM001721	Chr1:1922141:1923490:+	Phosphodiesterase
G1_GM001738	Chr1:1942976:1945048:−	c-di-GMP-specific phosphodiesterase class I
G1_GM001887	Chr1:2112072:2113784:+	c-di-GMP phosphodiesterase
G1_GM001963	Chr1:2197887:2200340:+	c-di-GMP phosphodiesterase A
G1_GM002029	Chr1:2274043:2275569:−	3′,5′-cyclic AMP phosphodiesterase CpdA T
G1_GM002209	Chr1:2470901:2473153:+	Phosphodiesterase
G1_GM002415	Chr1:2714768:2716021:+	Phosphodiesterase
G1_GM003033	Chr1:3405300:3405869:+	Type I phosphodiesterase
G1_GM003125	Chr1:3504090:3505742:+	Type I phosphodiesterase
G1_GM003196	Chr1:3576332:3577663:−	Diguanylate phosphodiesterase
G1_GM003204	Chr1:3587564:3588784:+	c-di-GMP-specific phosphodiesterase class I
G1_GM003579	Chr1:4007203:4008258:−	Glycerophosphodiester phosphodiesterase
G1_GM003053	Chr1:3427595:3428083:+	Phosphotriesterase family

**Table 2:** Molecular docking parameters of MPD with DDVP.

Protein	Substrate	Bond	Length (Å)	Kd (μM)	ΔG (kcal/mol)	Molecular-Interaction Energy (kcal/mol)	Electrostatic Potential Energy (kcal/mol)
MPD	DDVP	Hydrogen bond	2.60	144.15	−5.24	−6.25	−1.01

**Table 3:** Comparison of reported DDVP-degrading strains with strain G1.

Strains	Concentration (mg/L)	Time (h)	Degradation Rate (%)	DT_50_ (h)	Reference
*Flavobacterium* YD-4	400	48	60.89	15.60	[24]
*Lactobacillus plantarum* LAB	50	24	100	/	[28]
*Pseudomonas stutzeri* SMK	0.05	144	80	/	[18]
*Pseudomonas* AUG12	100	140	/	/	[25]
*Rhodobacter sphaeroides* EBL0706	400	12	98	/	[29]
*Ochrobactrum* sp. dichlorvos-1	100	24	100	/	[26]
*Halophilic* bacteria TL4	32.99	/	/	3.10	[27]
*Halophilic* bacteria T10/1	32.99	/	/	4.47	
*Nocardia mediterranei*	10	72	100	/	[30]
*S. acidaminiphila* G1	20	0.5	100	0.056	This study

## Data Availability

Data will be made available on request.

## Supplementary Materials

The supporting information can be downloaded at: https://www.mdpi.com/article/10.3390/ijms26199572/s1.

## Abbreviations

The following abbreviations are used in this manuscript:

DDVP	Dichlorvos
CFU	Colony-forming units
DMPP	Dimethyl phosphate
OPs	Organophosphorus pesticides
WHO	World Health Organization
EPA	Environmental Protection Agency
LB	Luria–Bertani
MPS	Massive parallel sequencing
PCR	Polymerase chain reaction
UPLC	Ultraperformance liquid chromatography
MPD	Methyl parathion hydrolase
