# Supplementary material for: Efficient Hydrolysis of Dichlorvos in Water by Stenotrophomonas acidaminiphila G1 and Methyl Parathion Hydrolase
Source: Int J Mol Sci. 2025 Sep 30;26(19):9572. doi: 10.3390/ijms26199572 (PMC12524942; doi:10.3390/ijms26199572)
Supplement: Supplementary file 1 [file ijms-26-09572-s001.zip › ijms-3833604-supplementary.pdf]

Supporting Information

This file includes:

Figure S1. (A) Extraction chromatograms, (B)mass spectra of DMPP and (C)DDVP at different degradation time by strain G1.

Figure S2. (A) Circle genome diagrams of G1 bacteria; (B) Functional classifications of genes in strain G1based on the COG database; (C) Annotation and analysis of genome from strains G1

Figure S3. Protein sequence comparison of degrading enzyme MPD with MBL and MPD.

Table S1. The MS/MS parameters for DDVP and its metabolites.

Table S2. The primers used for point mutation

Table S3. Degradation kinetics parameters of DDVP by strain G1 in different concentration.

Table S4. Degradation rate of other pesticides by strain G1.

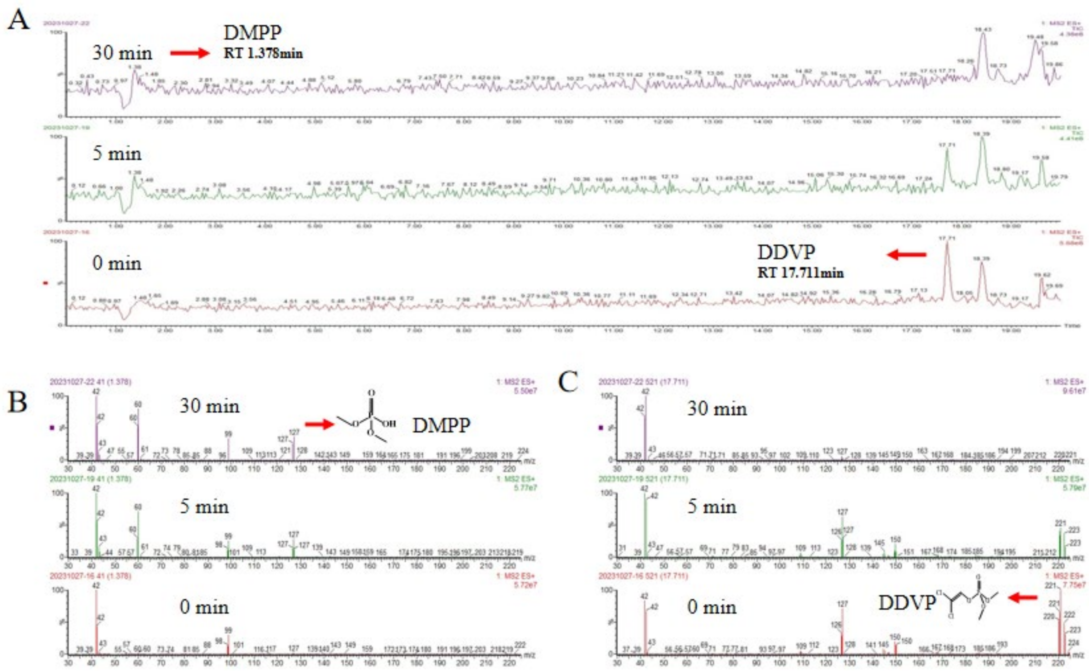

Figure S1. (A) Extraction chromatograms, (B) mass spectra of DMPP and (C) DDVP at different degradation time by strain G1.

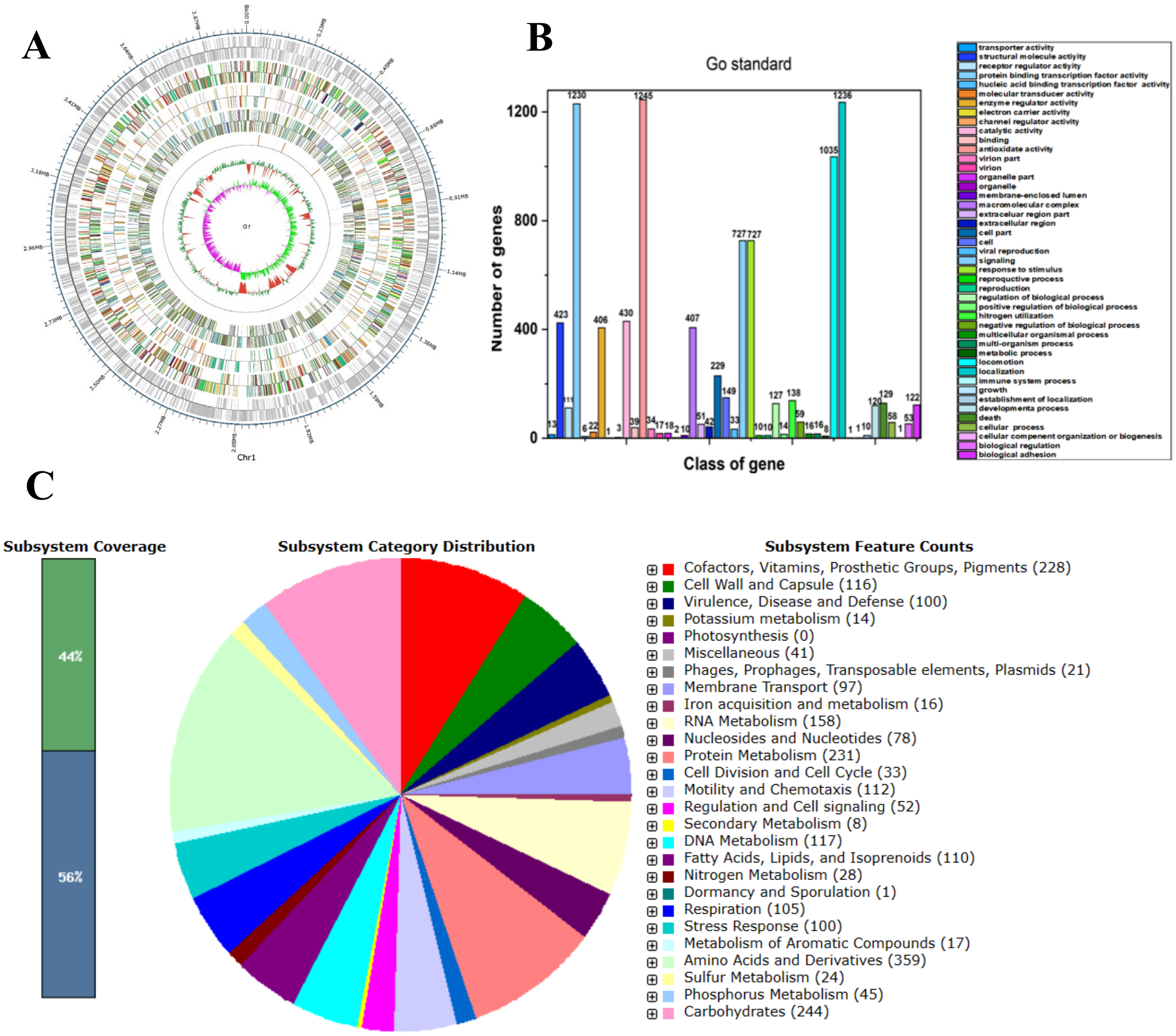

Figure S2. (A) Circle genome diagrams of G1 bacteria; (B) Functional classifications of genes in strain G1based on the COG database; (C) Annotation and analysis of genome from strains G1

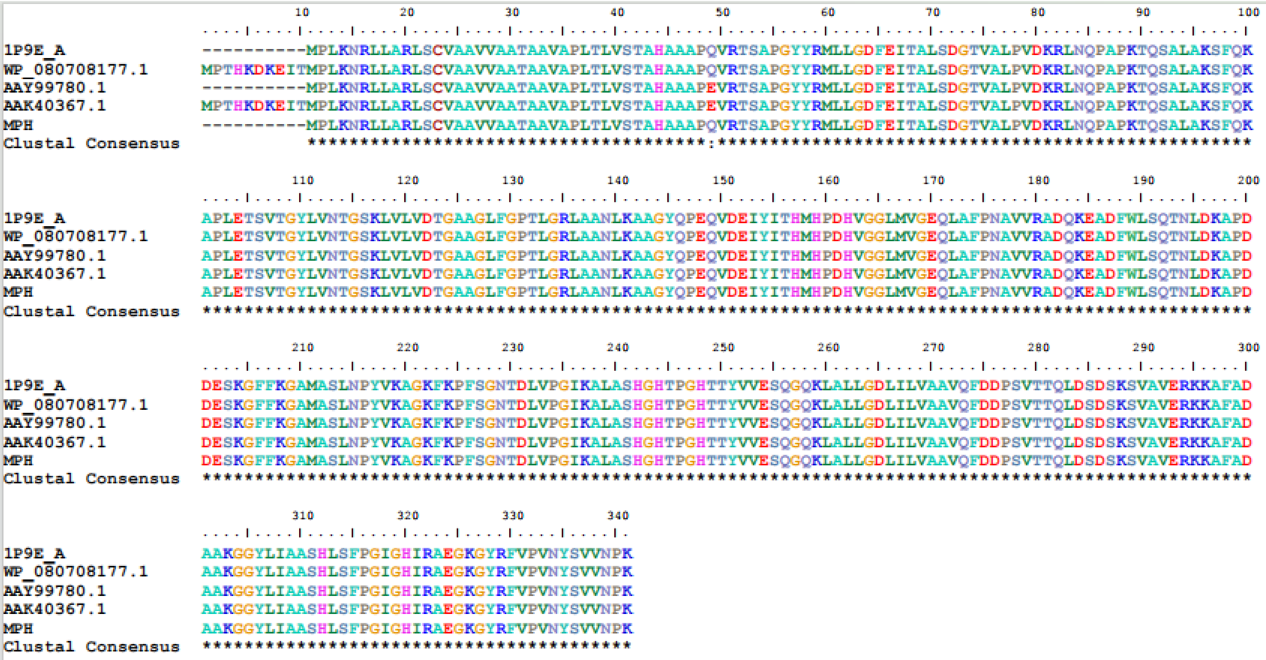

Figure S3. Protein sequence comparison of degrading enzyme MPD with MBL and MPD.

Table S1. The MS/MS parameters for DDVP and its metabolites.

Features	Chromosome 1
Size (bp)	4,016,985
G+C%	69
L50	1
Number of Contigs (with PEGs)	1
Number of Subsystems	421
Number of Coding Sequences	3674
Number of RNAs	71

Table S2. The primers used for point mutation.

primers	sequences
Asn-F	aagggctaccgtAATgtgccggtgaactactcggtc
Asn-R	acATTacggtagcccttgccttcg
Met-F	aagggctaccgtATGgtgccggtgaactactcggtc
Met-R	acCATacggtagcccttgccttcg
Arg-F	aagggctaccgtCGTgtgccggtgaactactcggtc
Arg-R	acACGacggtagcccttgccttcg

Table S3. Degradation kinetics parameters of DDVP by strain G1 in different concentration.

Concentration（mg/L）	Degradation kinetics equation	k (min^{-1})	DT_{50} (min)
200	$C = 186.90e^{-0.038t}$	0.038	18.24
500	$C = 465.34e^{-0.015t}$	0.015	46.20

Table S4. Degradation rate of other pesticides by strain G1.

Pesticides	Degradation rate of 20 mg/L OPs in 24 h
methyl parathion	100%
parathion	100%
chlorpyrifos	100%
fenitrothion	100%
phoxim	100%
triazophos	94.53±0.11%
profenofos	59.97±0.85%
thiram	21.7±1.21%
